# Can we trust measures of trust? a comparison of results from open and closed questions

**DOI:** 10.1007/s11135-021-01250-3

**Published:** 2021-10-06

**Authors:** Anna Brosius, Michael Hameleers, Toni G. L. A. van der Meer

**Affiliations:** Amsterdam School of Communication Research, University of Amsterdam, Amsterdam, The Netherlands

**Keywords:** Closed questions, Credibility, Media trust, Open questions, Survey research

## Abstract

Many public opinion surveys compare trust in a number of different information and (mediated) knowledge sources, typically using closed questions with a set of answer categories that are imposed by researchers. We aim to validate these categories by quantitatively comparing survey responses about trustworthy sources using open and closed questions, and by qualitatively analyzing the open answers. The results show that answer options typically used for closed questions in academic research are generally valid and closely match categories that respondents come up with unprimed. In some cases, answers to open questions can be non-exhaustive, particularly when sources are considered trustworthy but are not salient for respondents. Open questions, however, may still be useful for exploratory research or more detailed investigations of media diets on the outlet-level. Qualitative approaches to open questions can also give more insight into motivations for distrust, e.g. perceptions of inconsistency or a fundamental rejection of the shared factual basis of an issue. In addition, our results indicate that respondents’ interpretation of answer categories may change reported levels of trust: those that think of more specific outlets tend to report higher general media trust. This study provides new insights into how question design, and particularly the choice of answer options, may influence reported levels and sources of trust, and how qualitative and quantitative approaches to trust measurement can be combined.

## Introduction

A certain degree of trust in the news media and other sources of information and knowledge, such as political actors or expert sources, is fundamentally important in democracies; even more so during crises with higher levels of uncertainty and media dependency. During health crises, such as the outbreak of COVID-19 in 2020, media and political institutions play an essential role in providing citizens with information. At the same time, governments and health organizations depend on citizens to trust the information they distribute, and to comply with the interventions they propose. While declines in trust can be a response to official sources or media systems that are not able to provide accurate information, a lack of trust in (reliable) health information may be associated with undesirable outcomes—such as resistance to necessary behavioral adjustments (see e.g. Oksanen et al. 2020).

Given the importance of trust as a concept in communication science and other social sciences, its measurement deserves to be continually assessed – and updated to match changes in the information ecology. There have been numerous debates about trust measurement in several disciplines (see e.g. Kohring and Matthes 2007; Prochazka and Schweiger 2018 for the case of media trust). Given that most quantitative assessments of trust in information sources use closed questions with pre-determined answer categories, there may be a discrepancy between sources that citizens find relevant and sources that are deemed relevant by researchers, which respondents are then asked about in surveys. In part, these decisions on survey design are part of longer research traditions, that may not have been initially developed for today’s high-density and digitized information environment. This could mean that widely used measures of trust are not exhaustive or not precise–potentially overlooking sources of information that citizens find trustworthy. Conversely, presenting citizens with sources that they do not find important may result in invalid assessments of trust: they may rate sources as (un)trustworthy, even though they are unfamiliar or irrelevant to them. In order to test the validity of empirical assessments of trust in information sources, we need to take into account the overlap and potential discrepancies between existing trust measures and citizens’ unprimed trust assessment of sources. Do citizens understand trust in similar ways as envisioned in extant research, and, if not, for which elements do discrepancies arise?

We aim to answer this question by systematically analyzing respondents’ answers to open-ended questions about (un)trustworthy sources and comparing them to the results from closed questions that are in line with existing measures of trust used in the field. Specifically, we rely on survey data collected among citizens in Germany, the Netherlands, the UK, and the US, who were asked to indicate their trust in different sources during the 2020 COVID-19 outbreak. This particular health crisis was also dubbed an “infodemic” by the World Health Organization (WHO 2020) and academic literature (e.g. Nielsen et al. 2020) – highlighting the important role of public trust in the information provided by the news media and other institutions, like national governments, politicians, or health organization. It also presents a particularly interesting setting for measuring trust in information sources, as there was both a lack of knowledge and information, especially in the early phases of the outbreak, and later on a large amount of contradictory or even false information, as well as extensive discussions about the prevalence of disinformation. The results of this study provide new insights into how question design, and particularly the choice of answer options, may influence reported levels and sources of trust and whether open answers by respondents match with answer options that are typically used by researchers. The results show that there is a large overlap between sources that are mentioned by respondents in open questions and the closed answer options provided in surveys. Conclusions drawn from the two approaches are generally similar, though not identical, which provides support for the validity of widely used trust measurements.

## Theory

Trust, at its fundamental core, means expecting that a person or institution will fulfil one’s expectation towards them (Baier 1986). Trust in the media reflects citizens’ evaluations of the media, with a particular emphasis on the credibility of media information (Kohring and Matthes 2007; Tsfati and Cappella 2003). Political trust, situated between diffuse support for the political system and specific support for incumbents (Hetherington 1998), is a key indicator for citizens’ general support of politics. Credibility of the information that these actors provide is not often explicitly considered to be at the core of trust in them; though it is plausible to assume that a lack of credibility could damage trust in political actors, similar to how misconduct damages trust (van Elsas et al. 2020). In addition to political and general media trust, which are positively correlated (Ariely 2015; Köhler and Otto 2018), citizens may also trust a number of other sources of information, such as experts, specialized (non-)governmental organizations, platforms, or other people. Appelman and Sundar (2016) distinguish between credibility of the message, the source, and the medium. In this manuscript, we focus primarily on trust in sources, but build on literature on trust more generally.

Trust in politics and media typically grows in times of crisis, such as in the aftermath of terror attacks (Dinesen and Jaeger 2013). Due to heightened information needs caused by a crisis, media dependency and consequently media use increases (e.g. Hindman and Coyle 1999; Lowrey 2004). Similarly, the rally-around-the-flag effect (Mueller 1970) explains increased approval of political leaders during times of crisis. These two phenomena also occurred during the initial outbreak of the COVID-19 pandemic. For example, news use and political trust substantially increased in the initial phase of the outbreak (Esaiasson et al. 2020; Nielsen et al. 2020). However, for example in the United Kingdom, news avoidance increased after the initial increases in news use (Kalogeropoulos et al. 2020). Similarly, trust in media and the government as sources of information about the coronavirus declined again between April and May 2020 (Fletcher et al. 2020). Even though this effect may have been exacerbated by the specific domestic circumstances, it does indicate that positive effects of crises on news use, as well as media and political trust are not necessarily durable. Specifically, a notable number of citizens also has developed concerns about misinformation regarding COVID-19, both from politics as well as news organizations (Fletcher et al. 2020).

Institutional trust is generally associated with increased compliance with political decisions (Marien and Werner 2019). In the context of infectious diseases, trust can therefore be a crucially important factor in increasing compliance with containment measures. Countries with higher levels of institutional trust had lower mortality rates in the COVID-19 pandemic (Oksanen et al. 2020). Evidence from previous health crises points to the potential explanatory mechanisms. For example, Rubin et al. (2009) find that those who trust the government were more likely to follow behavioral recommendations during the swine flu; those with higher confidence in local health authorities were also more likely to do so during the SARS outbreak (Tang and Wong 2005). During the Ebola outbreak, reduced trust in government and belief in misinformation were associated with lower levels of adoption of preventative behaviors (Blair et al. 2017; Vinck et al. 2019). In the context of COVID-19, Nielsen et al. (2020) show that citizens rate news organizations and their government (with exception of some countries) as relatively trustworthy. Health organizations and scientists, doctors, and experts are highly trusted. Other citizens and politicians, as well as social media platforms, in contrast, are less trusted.

Information seeking is generally a situational and fluid activity guided by the perceived quality of information and trust in the sources providing the information. The proliferation of information channels and sources available to audiences as well as the wide variety of factors affecting information-seeking activities, make the concept of trust in information sources exceptionally complex to measure. Accordingly, survey methodology in general might only be able to capture ‘a snapshot in time’ of people’s perceived trust in information sources or information seeking behavior (Boyd, 2004). Within the context of a public-health crisis, this study sets out to explore trust in sources primarily by understanding where audiences turn to for information they find trustworthy. Therefore, we rely on a relatively broad definition of information sources to gain insights into which sources people tend to trust (and recall) in a situation characterized by high uncertainty and the absence of conclusive information. This broader definition includes information sources, institutions, and channels, ranging from knowledge sources, those producing the knowledge/information – e.g., the World Health Organization (WHO), experts, governmental actors –, to information sources, who gather and distribute the information in their function as channels or platforms – e.g., news media, social media, peers.

Here, we should also note that sources of knowledge and sources of information consulted by citizens are not always easy to distinguish. The WHO, experts or scientific institutions may produce knowledge on COVID-19 from their role as independent sources of knowledge. However, citizens may mostly learn from these sources via the media channels they select – for example TV news, social media platforms or interpersonal communication. Thus, official information is oftentimes mediated via other information channels.

While there is some consensus that institutional and media trust can play an important role for the effectiveness of health-related communication, there is more debate about its measurement. In large-scale surveys, such as the Eurobarometer or European Social Survey, institutional trust as an underlying latent construct (Marien 2011) is commonly measured using a number of questions that ask respondents how much they trust a number of specified institutions, such as the government, parliament, or the police. Institutional or political trust is thus assumed to be a one-dimensional construct (e.g. Hooghe 2011), even though others have argued that political trust is a multi-dimensional construct and should be measured as such (e.g. Fisher et al. 2010).

One-dimensional scales are also used to measure trust in different types of media (e.g. newspapers, TV, or radio), including in the Eurobarometer and the World Value Survey (see also e.g. Hopmann et al. 2015; Newman et al. 2019). These scales are particularly well-suited to compare levels of trust in different institutions and sources, but are also used as additive indexes (e.g. Hopmann et al. 2015). However, there are also a number of alternative measurements of general trust in the media, that account for the multi-dimensional nature of media trust (Kohring and Matthes 2007; Prochazka and Schweiger 2019). In addition, there are mixed approaches; Frewer and Miles (2003), for example, assess trust in different information sources using multiple items for each source.

These different schools of thought have one thing in common; they typically measure trust using closed questions, often with a pre-defined set of institutions to rate. Open questions are rarely used to identify sources of information that citizens deem trustworthy. This raises the question whether the selection of sources that is offered to respondents in these questions is congruent with the sources perceived as (un)trustworthy by the audience. This is particularly the case for one-dimensional measures that compare trustworthiness of multiple sources. Existing measures may not sufficiently consider today’s high-choice media environment, where news diets can consist of a mixture of traditional and alternative news sources, as well as non-professional social media sources (i.e., likeminded citizens, community or group pages, friends and family) that inform citizens on important issues. This particular question regarding the validity of the measuring of trust in information source may be assessed by comparing people’s answers to open versus closed questions to reveal discrepancies.

### Open-ended vs. closed questions

In general, there are two main reasons to use open questions, as opposed to closed questions: They reveal what respondents think of spontaneously and, maybe more importantly, reveal responses that are not biased by the given answer options (Schuman and Presser, 1996). Open-ended questions, when placed before closed questions that tap the same constructs, do not prime cognitive or affective schemata related to the concept of interest. In some cases, biases introduced by closed questions can be substantial and changes results of both descriptive and predictive analyses (Kealy and Turner 1993; Schuman and Presser, 1996). For example, studies found that they lead to different conclusions about patient satisfaction with doctors (Marcinowicz et al. 2007) or alcohol consumption (Ivis et al. 1997). However, there is also research that suggests that open-ended questions do not always improve predictive analyses of some outcomes (Friborg and Rosenvinge 2013). Open-ended questions come with their own challenges: Measurement error can be introduced while the answers are coded (Schuman and Presser 1996) and open-ended questions are left unanswered more frequently (Friborg and Rosenvinge 2013; Ivis et al. 1997). The usefulness of the more labor-intensive open-question format depends greatly on the specific research question one is trying to answer.

In the case of trust in media and politics, using open-ended questions may give us better insight in sources that respondents find trustworthy and think of spontaneously, without introducing bias through the answer options. To our knowledge, there is no previous research comparing the measurement of trust in media and politics using open and closed questions. However, there is evidence that pre-determined answer options can bias reported levels of trust; for example, trust in a supra-national political institution may be reduced when a non-trusted national institution is asked before within the same question block (Brosius et al. 2020). At the same time, respondents likely do not (all) think in the same categories as researchers designing survey questions. When asked about trustworthy sources in an open format, they may not think of certain options, even though they do find them trustworthy.

Therefore, if we are simply interested in comparing levels of trust in institutions that were selected for theoretical reasons, closed questions are the most useful format. If, however, we are interested in whether our pre-determined answer options actually line up with what respondents typically think of when asked about trustworthy information sources, open-ended questions are able to show us if we are overestimating the importance of some sources and neglecting other sources. At the very least, to validate existing scales and update them in new media realities, a comparison between open-ended and closed-ended measures of trust would allow us to (1) see to what extent unprimed answers on trust perceptions are congruent with traditional scales of media trust and (2) if there are additional sources of (dis)trust that are not captured in existing operationalizations. Thus, we ask the following research questions: *RQ1: To what extent do the sources of trustworthy information named by respondents in response to an open question overlap with typical answer options for closed questions?* and *RQ2: Do substantial conclusions differ if we use open vs. closed questions?* Different substantial conclusion could, for example, include which sources are identified as most or least trustworthy using different research approaches.

Furthermore, answer options also influence overall reported levels of media trust: Daniller et al. (2017) show that respondents report higher levels of trust when they are asked about specific media outlets (e.g. the New York Times), rather than the media in general. In closed questions, asking about generalized categories (e.g. *newspapers in general)* can be more practical than asking about specific outlets. However, Daniller et al. (2017) argue that such questions were initially designed when there was a much smaller number of mainstream media outlets that had important roles in shaping public opinion. Nowadays, citizens can select news outlets from a much larger and considerably more diverse media environment, which means that asking about “the media” or general information sources, like TV, online news, or newspapers, could mean very different things to different respondents. The data collected for this study allows us to test two things: First, we can see how many respondents name specific rather than general information sources. Second, we can see if those respondents that think of specific sources in open questions also have higher levels of media trust (measured on a traditional closed scale). We ask *RQ3: Do citizens that think of specific media outlets in open questions report higher general media trust?*

### Ecological validity and exhaustiveness of trust measures

 In digital information settings, characterized by high levels of choice (Waisbord 2018), and more relativism towards empirical evidence and expert knowledge (Van Aelst et al. 2017), closed-ended measures of trust may not be fully exhaustive or ecologically valid. News consumers may not always be certain whom to trust or could experience systemic distrust in all sources. These dimensions of (dis)trust in today’s media environment may not be captured in current closed-ended questions since respondents cannot answer along those lines easily. In addition, an in-depth analysis of open answers allows us to explore information that respondents volunteer unprompted, which may uncover potential issues with the measurement of trust in sources.

To this end, we rely on an additional qualitative analysis of the open-ended answers. Although the data was not collected for this purpose, inductive analyses allow us to arrive at a more comprehensive assessment of the exhaustiveness of existing scales, and the relevance of the current media ecology and crisis as a context in which people have to assess the trustworthiness of information. Based on current literature that has indicated increasing relativism toward the universal and fixed status of facts (Van Aelst et al. 2017; Waisbord 2018), and uncertainty in trust and information surrounding crisis times such as the COVID-19 pandemic (Nielsen et al. 2020), we regard *uncertainty*, *distrust,* and *factual relativism* as the key sensitizing (direction-giving) concepts of our qualitative endeavor. To inductively explore the exhaustiveness and ecological validity of the closed-ended trust measurements, we introduce the following research question *(RQ4): Do the open-ended answers indicate issues with the exhaustiveness and ecological validity of traditional, closed measures of trust in the current information ecology?*

## Method

The survey data were collected by an international panel company (Dynata) on March 19th and 20th, 2020. In addition, the open-ended questions were subject to a content analysis. Respondents were recruited in the United States, the United Kingdom, the Netherlands, and Germany. The survey was translated from English to Dutch and German by native speakers of the respective languages. In total, 1,912 respondents fully completed the survey. 185 of the 1912 respondents gave non-sensical answers to the open question and were therefore excluded, resulting in a total sample size of *N* = 1,727. Table 1 provides an overview of the individual sample composition for each country regarding number of respondents, gender, age, and education. As stated above, during data collection some governmental measures were implemented but varied across region or state.

**Table 1:** Sample composition and number of deaths/infections across countries

		US	UK	NL	GER
N		349	521	403	454
Gender	Women	74.14%	52.60%	54.59%	49.45%
	Men	25%	47.40%	45.41%	50.33%
	Other	.86%	0%	0%	.22%
Age		50.31 (13.7)	46.71 (12.49)	42.52 (13.71)	45.29 (13.41)
Education	Low	16.95%	17.53%	14.39%	32.60%
	Medium	57.76%	46.82%	38.71%	39.21%
	High	25.29%	35.65%	46.90%	28.19%

## Measures

At the start of the survey, we measured general media trust by asking respondents how much they agree or disagree with three statements on a scale from 1 (fully disagree) to 7 (fully agree): ‘I think you can trust the news most of the time’, ‘I think you can trust most news organizations most of the time’, and ‘ I think you can trust journalists most of the time’. This is a shortened version of the media trust scale used in the Reuters Institute (e.g. Newman et al. 2016). Respondents were then introduced to the focus of the study on the new coronavirus (SARS-CoV-2). It was mentioned that the virus was among the most pressing issues in the news today and received a lot of attention in all countries across the globe. It was stated that, in the questions that followed, respondents would be asked about their behavior related to the new coronavirus and its presence in the media. Next, respondents were first presented with an open-ended question about their trust in information sources, followed by a closed question that inquired about the same concept. Sociodemographics like age, gender, and education were measured at the end of the survey. The survey also included other questions that were part not part of this research project.

*Open-ended question source trust* The first coronavirus-related question that respondents answered was an open question about trustworthy sources. To introduce the topic, the questionnaire stated that respondents had probably received a lot of information about the coronavirus over the last few days. Respondents were then instructed to list five sources that they find most trustworthy for information regarding the coronavirus, ordered from most to least trustworthy. The instructions further illustrated that such sources could, for example, be any media sources or people they know.^1^

*Closed question source trust* After answering the open-ended question, respondents were directed to the next page that showed a battery of information sources. In the instructions they were asked to rate how trustworthy they find the sources listed when regarding information about the coronavirus. Respondents rated these sources on a Likert-scale from 1 (very untrustworthy) to 7 (very trustworthy). The exact wording of the closed question was: “We also want to ask you to rate how trustworthy you find the sources listed below when it comes to information about the coronavirus.” The following sources were listed: government, head of state, WHO, national health organization, newspapers, TV, radio, Twitter, Reddit, Instagram, Snapchat, TikTok, YouTube, friends and family, colleagues and acquaintances, and strangers. These choices were based on previous research (e.g. Eurobarometer surveys (European Commisson, 2018) that ask about printed newspapers and news magazines, television, radio, online newspapers and news magazines, video hosting websites and podcasts, and online social networks and messaging apps); the health organizations were added due to their topical relevance (similar to e.g. Fletcher et al. 2020 and Nielsen et al. 2020 who asked about scientists/doctors/health experts, national and global health organization, news organizations, the government, people one knows or does not know, and politicians).

### Coding of open-ended answers

The open-ended answers were manually coded. A codebook was developed to determine whether the sources listed in the *closed* questions were mentioned in the *open* answers, whether a specific media outlet was mentioned, or whether they mentioned social media or news in general as a trustworthy source. The coding of the open answers was conducted by three researchers involved in the project, after a training session. Before the start of the actual coding, we conducted a test of intercoder reliability on 50 randomly selected open answers. Intercoder reliability was assessed using Krippendorff’s alpha, with values ranging between 1 and 0.73, with the exception of one variable which was dropped from the analysis due to its lower reliability at α = 0.66. See Appendix A for the exact values for all variables.

### Exploratory qualitative analysis

For the inductive qualitative analyses of the open-ended responses, we rely on the stepwise coding procedures of the grounded theory approach (Charmaz 2006). Two coders first read and re-read all open answers with the sensitizing concepts in mind. After this first round of familiarization, they selectively arced open answers that reflected relevant variety and insights on the concepts of interest (i.e., distrust, uncertainty or relativism). During this first step, these segments were assigned descriptive labels (open codes). After the open coding process, the unique codes were merged to higher-order themes, grouped or restructured in a more analytic and less context-bound manner (selective coding). We looked for main themes and variations on themes that best described the variety on our sensitizing concept. We finally looked for relationships between the emerging themes and categories (axial coding).

Traditional assessments of validity and reliability used in textual quantitative coding are not transferable to the inductive and interpretative nature of qualitative data. We therefore relied on peer debriefing and constant comparison as validity and reliability checks suited for the interpretative and rich nature of qualitative data. Specifically, the full corpus was coded by two researchers, who discussed every disagreement until reaching agreement. Emerging codes and themes were constantly compared to earlier codes and themes to check for saturation and consistency in coding. A third researcher who was not involved in the coding process assessed and discussed all coding procedures. During this step of peer debriefing, complete agreement on the procedures and the data reduction processes was achieved (the three coding steps and the resulting themes from the raw data).

## Results

### Quantitative analyses

RQ1 concerns similarities between the answers to open questions and closed questions regarding trustworthy sources of information. Sources which we had not included in the closed question but were often named by respondents include healthcare workers (e.g., doctors and nurses, named by 9.1% of respondents), local news (7.6%), and Facebook (5.0%). This indicates that these may be items that could have been relevant to add as closed questions. Some respondents also gave general answers, such as news websites in general (7.8%), the news in general (5.7%), and social media in general (2.3%); however, these items could be covered by more specific closed questions. Besides these sources, 78.8% of respondents do not mention any other sources that were not also part of the closed questions. That means that the options for closed questions generally cover sources of information that are relevant to the majority of respondents, even though there are potentially relevant additions.

**Fig. 1:**
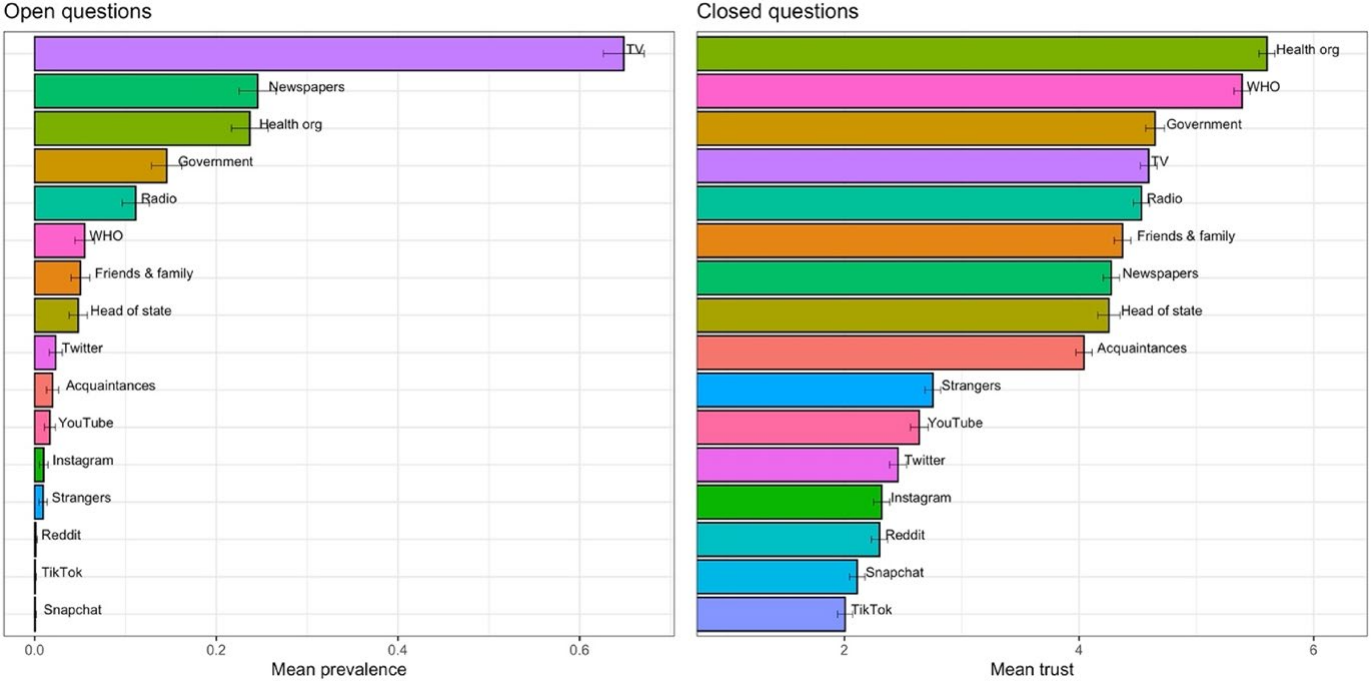
Mean scores for open and closed trust questions

Turning to RQ2, the descriptive results illustrated in Fig. 1 show that barely any respondents name specific social media sources, such as Twitter, YouTube, Instagram, Snapchat, TikTok, and Reddit in the open questions. Respondents also rarely mention colleagues, acquaintances, or strangers. This corresponds to lower trust levels in these sources reported in the closed questions; i.e. sources that are barely mentioned also tend to score lower on trustworthiness. In this regard, conclusions drawn from open and closed questions are similar when it comes to the order of most to least trustworthy sources. However, the differences may seem less drastic in closed questions; for example, while virtually no respondents name social media platforms like TikTok or Snapchat, this is not reflected in a near-zero (albeit low) trust score in the respective closed questions. Furthermore, the order of trustworthiness does change for some sources. Most notably, health organization like the WHO or the respective national health organizations (RKI, RIVM, CDC, and NHS), are rated most trustworthy in closed questions; however, they are not most often named in the open questions. Instead, respondents most often name TV stations (or their websites and apps) as trustworthy sources. This may indicate that respondents typically obtain their information from traditional news outlets (which makes them more salient), even though they deem the information of health organizations most trustworthy. Another interesting pattern is that, even though the WHO and national health organization are rated as similarly trustworthy in closed questions, the WHO is named considerably less often than national health organizations by respondents; likely because they turn more to the national health organizations to obtain information. Similar patterns (albeit less pronounced) can be observed for the government and friends and family (who are seen as trustworthy but may not be a very salient information source).

In addition to comparing the descriptive conclusions from open and closed questions, we also examined whether respondents that named a source in the open questions reported higher levels of trust in that source in the closed question. Table 2 shows these t-tests. For many sources, including the national government, heads of state, the WHO, national health organizations, newspapers, TV and radio programs, and friends and family, the average trust indicated in closed questions is about one point higher for those that named the same source in the open questions, indicating response consistency. We see even larger discrepancies in trust in social media (and thus response consistency) between respondents who did or did not name social media outlets in the open questions. Twitter, Instagram, and YouTube have relatively low trust scores in general, but do score high among the small number of respondents who did name them as trustworthy sources in the open questions. We did not conduct t-tests for Snapchat and TikTok, which were each only named by one respondent.

**Table 2:** Mean trust reported in sources that were named or not named in open questions

Source	Not named in open question *M* (*SD*)	Named in open question *M* (*SD*)	t-statistic	df	p value
Government	4.47 (1.69)	5.67 (1.34)	− 12.61	399.55	< 0.01
Head of state	4.18 (2.00)	5.67 (1.73)	− 7.63	93.512	< 0.01
WHO	5.32 (1.46)	6.48 (0.82)	− 12.63	131.31	< 0.01
Health organization	5.39 (1.47)	6.28 (0.99)	− 14.11	1016.9	< 0.01
Newspapers	4.06 (1.48)	4.92 (1.27)	− 11.68	826.59	< 0.01
TV	3.92 (1.65)	4.95 (1.28)	− 13.37	1006.6	< 0.01
Radio	4.44 (1.47)	5.23 (1.29)	− 7.86	256.9	< 0.01
Twitter	2.41 (1.50)	4.23 (1.72)	− 6.62	40.423	< 0.01
Reddit	2.29 (1.47)	5.50 (2.12)	− 2.14	1.0011	0.29
Instagram	2.29 (1.45)	4.35 (1.77)	− 4.79	16.215	< 0.01
YouTube	2.60 (1.57)	4.97 (1.24)	− 10.16	29.561	< 0.01
Friends and family	4.32 (1.49)	5.29 (1.40)	− 6.23	96.608	< 0.01
Colleagues and acquaintances	4.03 (1.47)	4.47 (1.38)	− 1.84	1724	0.07
Strangers	2.75 (1.42)	3.19 (1.47)	− 1.19	15.262	0.25

**Fig. 2:**
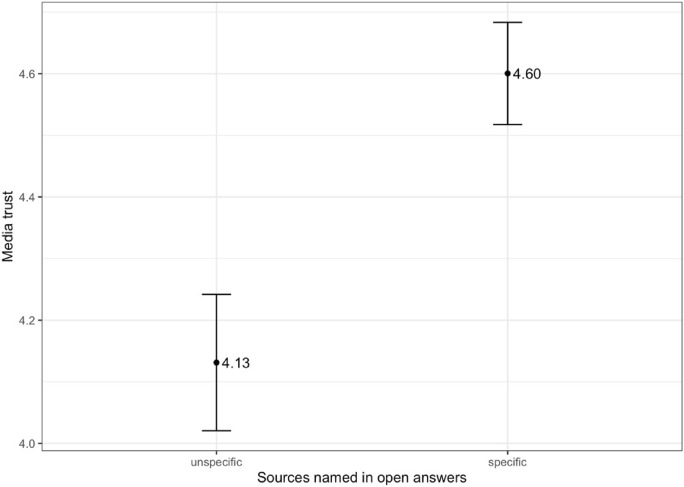
Mean media trust for respondents naming unspecific vs. specific outlets in open questions

Our results also show that over half of all respondents (52,8%) named at least one specific media outlet in the open questions. This means that a considerable number of respondents think of rather specific outlets when asked about trustworthy sources – and these might not be reflected in closed questions referring to broad media categories. Concerning RQ3, and as displayed in Fig. 2, the results show that those respondents that report higher general media trust tend to name more specific outlets. Even though we cannot be sure about respondents’ thought processes, this could indicate that respondents that have specific media outlets in mind when asked about trustworthy sources may also be more likely to think of those sources when they are asked about their general media trust, and thus report higher levels of trust in the media *in general.* Possibly higher levels of media trust could go hand in hand with a more nuanced evaluation of the information setting: While not all sources may be equally trustworthy, there are specific outlets that can be trusted.

### Qualitative analysis

After analyzing the open-ended responses using the stepwise Grounded Theory approach, we found four main themes: (1) changes in the information setting result in uncertainty and lower perceived trustworthiness; (2) inconsistency within or between sources makes it difficult to assess trustworthiness; (3) a rejection of the reality depicted by the media is connected to complete distrust; (4) information can be considered untrustworthy due to deliberate deception or alleged (political) motivations to mislead the public.

One of the most prominent themes is level of uncertainty people express in their credibility assessment. In the setting of conflicting evidence and changing opinions, people were unsure whom they could trust: “I trust so-called medical experts a little but in general I think nobody really knows anything for certain.” This also relates to the notion of inconsistency that many respondents experienced: “I don’t trust a lot of information because it is so conflicting.” Some respondents outlined that a boundary condition for trust is consistency between and/or within sources. When information changes over (a short period of) time, or when different sources tell different stories, respondents find it difficult to trust any information at all, or to judge trustworthiness. In this context of conflicting evidence and uncertainty, some respondents expressed the need to be skeptical: “I just listen to all of it and take what they all say and pick out what seems to be common in all the info I receive and go from there.” This points to media literacy in a context of high uncertainty. As one other respondent emphasized: “I cannot name five sources as most have to be thoroughly checked before being viewed as truthful.”

Although uncertainty, perceived inconsistency and the need for active verification could indicate ‘healthy’ levels of skepticism that are required to navigate a complex information setting, our analyses also point to two themes that more closely reflect system-level distrust. Some respondents did not name any trustworthy sources as they did not believe that COVID-19 was a real threat: “I think it’s a waste of time and it’s just like the flu.” Some respondents even expressed that they were being lied to: “Think it's lies, to make people think that it is virus, it's cold and flu season.” This shows that agreement on the givens of a situation is a boundary condition for trust in sources. Some also view media and governmental information as deliberate deception: “Governments and media have a great deal to answer for in helping create and continually fuel this situation!” Others more specifically pointed to the political motives of information sources: “What I read in general news is all aimed at panicking the public with political intent.”

Together, the analysis of the open-ended answers shows that – although traditional closed question on trust are valid – there are themes related to skepticism and distrust that are not always captured in quantitative measures of media trust. We found that distrust can relate to different motivations and boundary conditions. We can summarize the results in three main clusters of distrust: (1) some news users may distrust information due to perceived inconsistency and unclarity, which they can compensate with critical media literacy skills; (2) others perceive certain (i.e., political or financial) motivations as underlying factors of untruthfulness and (3) a final group of news users point have a view on reality that is fundamentally different from the reality depicted in the mainstream media, which corresponds to a system-level rejection of information sources.

## Discussion

In order for citizens to make well-informed political decisions, they need to trust the institutions informing them, such as the government, news media, and non-profit organizations. Not surprisingly, there is an abundance of literature on trust and its operationalization in the social sciences. Although this literature has called for more precise and valid operationalizations of (media) trust, for example by introducing multidimensional scales that move beyond single-item evaluations (e.g., Kohring and Matthes 2007), we know markedly little about the ecological validity of scales that compare trust in sources: Do the categories of trusted sources distinguished in quantitative survey research match citizens’ unprimed assessments of trust, and are the sources typically included in closed questions exhaustive and generalizable across different national settings?

Attempting to validate existing operationalizations of citizens’ trust in information in the setting of the COVID-19 pandemic, we collected survey data in four countries: the US, the UK, Germany, and the Netherlands. In this survey, we compared open-ended questions of trust to closed-ended questions and explored to what extent existing measures of trust are able to precisely and exhaustively capture the unprimed evaluations of citizens. Generally, our main findings call for optimism regarding the validity of these scales. Sources mentioned in the open-ended trust questions overall match the categories of the closed-ended questions. Conclusions drawn from answers to both types of questions are similar, showing that the conceptions of trust envisioned by citizens correspond to the categories of trust suggested by researchers.

However, there are some noteworthy discrepancies. First of all, in the closed questions, national and international health organizations were seen as most trustworthy. In the open questions, in contrast, TV was mentioned most often. This could indicate that, in open questions, trust is partially conflated with frequency of use and memory. TV may be mentioned as this is the most dominant source used by citizens, and therefore easily available when people are primed to think about trust. A potential limitation of open questions in that regard may be the failure to activate relevant categories in the minds of receivers. Even though people may trust some sources, they may not be cognitively accessible without a prime. This is particularly interesting in the case of health organization, such as the WHO or CDC, as many mainstream media sources rely on their information and simply act as a mediator between them and citizens.

Analyzing the open-ended questions, we found that many respondents did not think about trust at the level of the medium, but at the more specific level of sources, such as BBC News. Closed questions in empirical research, in contrast, often ask for trust in non-specific sources (i.e., TV news, newspapers, radio). In addition, the more people trust the media, the more likely they are to name specific sources. These findings indicate that, although traditional measures of trust are relatively accurate, they may not directly match the ways in which citizens themselves evaluate trust. Especially in the context of fragmented media diets and high-choice, citizens have more control over their media diets – and can avoid sources they distrust. In this setting, media trust measures that distinguish between a subset of specific sources for all media types may yield a more accurate assessment than generic trust measures. However, researchers should be aware that asking about specific outlets may prime respondents and result in higher levels of reported trust than asking about more general categories.

The qualitative analysis of the answers to the open-ended questions further revealed some motivations of those respondents who refused to answer the question as such – something that closed questions do not allow for. For example, inconsistency between or within sources, fundamental issue disagreement, and the perception of intentional deception motivated distrust. These more detailed insights may not be relevant for quantitative research endeavors interested in general levels of trust but could be meaningful for studies that aim to assess news users' perceptions in specific settings or specific populations of news users, for example in the context of conspiracy believes. As a practical recommendation, we suggest researchers to assess whether they are targeting a general audience or a more specific sub-sample when inquiring about trust.

It should be noted that we collected our data in the midst of a highly salient international health crisis. Media system dependency theory holds that – when societies face a situation of crisis or instability – the news media play an even more important role in providing citizens with trustworthy and accurate information (Ball-Rokeach and DeFleur, 1976). In a context such as the COVID-19 crisis, assessing trust levels is particularly important, among other things due to the relation between media trust and compliance with health regulations (Hameleers et al. 2020). Despite the importance of trust in crisis times, we know little about how our assessment of the validity of trust measures is generalizable to routine periods in which media dependency is lower. We asked people to indicate to what extent they trusted different sources related to information on the pandemic – more research is needed to be able to generalize our findings to other contexts.

The broad conceptualization of information sources applied in the current study limits us in determining whether trust levels and measures differ across certain types of sources. It is difficult to distinguish between what type of sources people trust intuitively, i.e. the results from open ended questions, where respondents are asked to think of sources themselves and the results from closed questions where a variety of predetermined information and knowledge sources are mentioned. For example, the recommendations and analyses produced by the WHO might only reach audiences via news media or other channels. Reported trust in news media might therefore also entail trust in the sources the news rely on and therefore indirectly include knowledge sources like the WHO. Moreover, we cannot differentiate between the specific features related to institutional trust such as the legitimacy of the goals, procedures, and capacity of a certain institution. For example, trust in news media and social media are now addressed in similar ways. Yet, news media might be trusted since they follow certain editorial procedures which lead to predictable and reliable information. In contrast, social media platforms generally do not follow such ‘gatekeeping’ procedures. However, people might indicate trust in such platforms because they trust specific institutions or individuals on social media due of their legitimate goals or information gathering procedures. Future research should make a clearer distinction when assessing the measures of trust in information sources, where, for example, in the open and closed questions respondents are explicitly asked about their trust in different type of sources (e.g., both knowledge and information sources) and what (predetermined) features of such sources makes them trustworthy.

Related to the limitation addressed above, the precise formulation of the open-ended question might have affected the comparison between the two types of measures. When asked about the most trustworthy sources in the open-ended questions, respondents might be less likely to think of knowledge sources than information sources (i.e., the news media or social media). Accordingly, part of the differences observed in the measures, especially for sources like the WHO or national health authorities, may be a product of the question wording. When answering the open-ended question, there might be a difference between which sources respondents trust versus which ones they recall without a prime (like in the closed question). Therefore, the comparisons made in the manuscript regarding what type of sources are recalled in the open-ended questions versus the ranking in the closed questions needs to be interpreted with the appropriate caution.

In our closed questions, we relied on a very basic measure of media trust that did not take into account the multidimensional nature of trust and credibility (e.g., Kohring and Matthes 2007). It may be the case that the correspondence between open and closed questions changes with a different conceptualization of trust as a multi-dimensional construct. Especially in an information setting characterized by mis- and disinformation and increasing concerns on both the accuracy and honesty of information (e.g., Van Aelst et al. 2017), it may be worthwhile for future research to validate multidimensional trust measures using open questions. Finally, even though we attempted to achieve samples representative of the populations in the four countries, this was not always possible; for example, the distribution of gender in our US sample is not representative. However, this is a less pressing issue, since we focus on comparing answers to different questions from the same people; however, it may impede the generalization of the results in some contexts. Despite these limitations, we aim to make a contribution to the trust literature by validating the most used assessment of media trust in an internationally comparative sample. Overall, although traditional measures may not capture citizens’ evaluations of trust related to specific media outlets, these measures can be regarded as a valid operationalization of general levels of trust in different sources of information. Even though the use of open questions is often warranted for specific research question, the high costs of analyzing the results may outweigh their benefits for research that aims to assess the trustworthiness of sources quantitatively.

## Appendix

See Table 3.

**Table 3:** Intercoder reliability, Cronbach’s alpha values

Var 1: Government mentioned	0.852	Var 2: Head of state mentioned	1
Var 3: WHO mentioned	1	Var 4: National health org. mentioned	0.962
Var 5: Newspapers mentioned	0.726	Var 6: TV mentioned	0.88
Var 7: Radio mentioned	0.846	Var 8: Facebook mentioned	1
Var 9: Twitter mentioned	1	Var 10: Reddit mentioned	1
Var 11: Instagram mentioned	1	Var 12: Snapchat mentioned	1
Var 13: TikTok mentioned	1	Var 14: YouTube mentioned	1
Var 15: Friends and Family mentioned	0.924	Var 16: Colleagues and acquaintances mentioned	0.936
Var 17: Strangers mentioned	0.771	Var 18: Local media mentioned	1
Var 19: Healthcare workers mentioned	1	Var 20: News in general mentioned	0.895
Var 21: Specific media outlets mentioned	0.926	Var 22: News websites mentioned	0.73
Var 23: Social media mentioned	0.895		

## Footnotes

1 The exact wording of the open-ended question was as follows: “Please list the sources that you find most trustworthy for information regarding the coronavirus. These sources could for example be any media sources or people you know.”.
